# Malarial Cachexia

**Published:** 1873-01

**Authors:** Charles P. Gordon

**Affiliations:** Dalton, Ga.


ATLANTA

### MEDICAL AND SURGICAL JOURNAL.

VOL. X.—JANUARY, 1873.—NO. 10.

### ORIGINAL COMMUNICATIONS.

### MALARIAL CACHEXIA.

BY CHARLES P. GORDON, M.D., Dalton, Ga.

The writer desires to present to the profession, through the medium of your very excellent journal, a few thoughts relative to a condition of system, which, from its frequency in almost every section of our country, is a subject of grave importance to every practising physician. And although no new theory in medical philosophy is intended to be advanced, yet we shall feel content if enabled to bring prominently and impressively before the profession a fundamental and controlling idea in the therapeutics of that state of system denominated malarial cachexia.

Probably no extensive region of our country escapes entirely the baneful influence of malaria: It prevails from the gilded east to where the "purple rays of day" fades away in the distant west, and from the great lakes in the north to the boiling, seething cauldron of the Gulf of Mexico in the south. Our own southern land is especially afflicted with the extensive prevalence of this subtile ærial poison.

We do not, however, propose to study the natural history of malaria, the physical laws that govern its production and development, or its essential nature, whether gaseous or solid, organic or inorganic, animalcular or of vegetable origin. These, although questions of paramount scientific importance, we leave to the investigation of abler observers. When we reflect that the blood of millions of human beings is liable to deterioration and impoverishment by the agency of this mysterious influence, it becomes a consideration of no minor importance to study the best therapeutic means of controlling successfully its effects, although we may have no definite knowledge of the essential nature of the cause itself. Should future scientific research reveal the true nature of this specific cause of periodic disease, then if we cannot neutralize directly the morbific agent itself, from a more definite knowledge of the etiology of this class of diseases, we may be enabled—*a priori*—to arrive at more practical and philosophic conclusions concerning their treatment; but in the present state of knowledge we must content ourselves with a process of reasoning—*a posteriori*—hoping not so much to comprehend the nature of the cause from the consideration of its effects, as to discover the remedial measures best adapted to the cure of those diseases, the ligitimate result of that cause operating upon the human system. It is unnecessary to enumerate the multiform types of periodic disease which recognize malaria as their specific cause. The various treatise before the profession are already exhaustive of the subject.

It is the object of this communication to consider only the chronic effect of malarial influence upon the system, called malarial cachexia, with the remedy offering the best prospect of cure.

Among the protean types of disease, the result of the chronic influence of malaria, probably none are oftener presented than chronic inflammation and hypertrophic enlargement of the spleen, the result of the frequent congestions to which that viscus is subjected in the ordinary attacks of intermittent fever. This organ, serving as a diverticulum for the blood during the internal congestion attending the cold stages of intermittent fever, is the subject of hypertrophy and histologic change from an interstitial deposit of matter foreign to its proper structure and materially interfering with its function, which, although not definitely demonstrated, is inferentially an important office in the process of hæmatosis. Nature is never guilty of the work of supererogation. The spleen doubtless performs an important function in the animal economy; and from the extensive distribution of blood to that organ and the great number of lymphatic vessels found within its structure, we conclude almost with certainty, that it performs an important part in the elaboration of the blood. Granting the truth of this theory, what would be the legitimate result of disease of this organ preventing or abolishing its important function? Obviously a deterioration and impoverishment of the blood; and such a result we invariably find in clinical experience. Enlargement of the spleen, popularly called ague cake, is a very frequent concomitant of chronic malarial disease.

The subjects of this disease, always the residents of malarial regions of country, are peculiarly noted for their wan, cachexic appearance, possessing an hydremic condition of the blood, consequent upon a reduction of the normal quantity of its plastic and corpuscular elements; the effect of the continuous operation of a poison directly impairing its integrity and producing disease in an organ essential in the process of sanguification, resulting in a general perversion of nutrition, and all the evil consequences conjoined in malarial cachexy. The blood is the life; and although probably the first morbific impression of malaria is upon this fluid, as it is inhaled into the lungs in the form of an ærial poison and conveyed through its medium to every tissue in the animal economy, impressing each in accordance with their several pathological susceptibilities; yet we hold that the deleterious effect upon the vital fluid is greatly enhanced by the involvement in disease of that organ, the integrity of which is so essential to its healthful constitution, and the maintenance of its normal standard. The blood being thus degenerated and devitalized by the continuous effect of malaria, what may we conclude from this view of the pathology of malarial cachexia as to the best therapeutic means to be adopted for its cure? Evidently the administration of that remedy best calculated to restore this fluid to its normal standard of health; and if asked what that remedy is, we answer with decision and emphasis: *the strong arm of iron.* It matters not what form of chronic malarial disease presents itself, whether chronic intermittent fever, neuralgia, or that general constitutional depravity known as malarial cachexy, while we wonld rely upon the sulphate of quinine to break the periodic concatenation, iron is the great hæmatopoetic remedy which is to revivify the blood and restore the patient to health and strength. Even in malarial hæmaturia or hemorrhagic malarial fever, that more recent manifestation of malarial disease in which the blood is prominently devitalized by the continuous action of the poison, with a general hemorrhagic tendency to the bronchial, gastro-intestinal and urinary mucous membranes, iron, in the form of the muriated, we would regard as the *great curative remedy.* Probably the strongest predisposing cause of hemorrhagic malarial fever is a chronic malarial toxæmia. The subjects of this formidable disease have generally suffered with some form of chronic malarial fever. May not malarial hæmaturia then be prevented by a timely resort to the use of iron? The object then should be to keep the systems of those suffering with chronic malarial disease thoroughly ferrated by the frequent exhibition of iron, not only as a curative measure for that condition itself, but as a preventive means for that pernicious form of malarial fevers, called malarial hæmaturia.

The writer would advise all sufferers with chronic malarial disease of any form to avail themselves, if practicable, of the natural preparations of iron as found in the chalybeate springs which abound in various sections of our country, which, besides affording the advantage of removal from the malarial influence, present iron in the form most readily assimilated and carried into the circulation. It seems, as prepared in the laboratory of nature, the preparation of iron far excels any form as yet presented to the profession. The medical chemist might profitably study the theory of the formation of these chalybeate springs, and present a more soluble preparation of iron than has yet been offered. We find deeply imbedded in the earth the carbonate of the protoxide of iron, entirely excluded from all atmospheric agency; the water permeating the earth and becoming charged with this form of iron, held in perfect solution, appears at its surface in the form of chalybeate springs; coming in contact with the atmosphere the carbonic acid gas is soon evolved, and the iron, converted by taking oxygen from the air into the insoluble sesqui-oxide, is deposited in the form of red sediment in the stream.

These chalybeate springs were resorted to in the primitive history of our country by the indians, to cure their malarial fevers; and we have observed from long experience that these natural preparations of iron excel in the cure of malarial cachexia all preparations by art—being held in perfect solution, the iron readily enters the circulation and restores the blood to a standard of health.

---

### GENERAL CLINIC ATLANTA MEDICAL COLLEGE.

SERVICE OF WM. ABRAM LOVE, M. D.,
*Professor of Physiology and Clinical Lecturer on Diseases of the Liver and Kidneys.*

CASE 5. *Rheumatism, Acute, with Intermittent Fever.*—J. M., æt. forty, colored, and a widow, an in-door laborer, was taken a week ago with a chill, followed by fever passing off with diaphoresis; at the same time she suffered from pain in the right knee and ankle joint, with heat, swelling, and an inability to move them without much suffering. Slight chills, followed by fever, have returned on alternate days, until the present. She ceased menstruating in May last; has suffered no inconvenience in this respect; tongue slightly coated; fauces somewhat discolored—of a yellowish tinge; bowels regular; urine acid; heart sounds normal; respiration good; and she suffers only with the chills and fevers, and pain in the knee and ankle. The joints, as you will observe, are very much swollen. She has been the subject of rheumatism before, the evidence of which is presented in the enlargement and stiffness of some of the finger joints of the right hand, as you see. We have, then, in this case to contend with rheumatism complicated with malarial poison; they are not at all incompatible with each other, nor is the treatment; we shall, therefore, recommend—

℞.—Quinia sulph., grs. vi.

Morphia sulph., gr. $\frac{1}{4}$, *ter die*—

To arrest the malarial fever and allay pain, a cholagogue cathartic (i.e., pil. cath. comp. U. S. P.) to evacuate the bowels and remove portal engorgement; to be followed by—

℞.—Pot. bicarb., grs. xxx, *ter die*—

